# Radiation dose-rate is a neglected critical parameter in dose–response of insects

**DOI:** 10.1038/s41598-022-10027-z

**Published:** 2022-04-14

**Authors:** Hanano Yamada, Vanessa S. Dias, Andrew G. Parker, Hamidou Maiga, Carina Kraupa, Marc J. B. Vreysen, Wadaka Mamai, Marc F. Schetelig, Nanwintoum S. Bimbilé Somda, Jeremy Bouyer

**Affiliations:** 1 Insect Pest Control Laboratory, Joint FAO/IAEA Centre of Nuclear Techniques in Food and Agriculture, Friedensstrasse 1, 2444 Seibersdorf, Austria; 2 Department for Insect Biotechnology, Justus-Liebig-University Gießen, Winchester Str. 2, 35394 Gießen, Germany; 3 Present Address: Roppersbergweg 15, 2381 Laab im Walde, Austria

**Keywords:** Experimental nuclear physics, Entomology, Biological techniques, Genetic techniques

## Abstract

Reproductive sterility is the basis of the sterile insect technique (SIT) and essential for its success in the field. Numerous factors that influence dose–response in insects have been identified. However, historically the radiation dose administered has been considered a constant. Efforts aiming to standardize protocols for mosquito irradiation found that, despite carefully controlling many variable factors, there was still an unknown element responsible for differences in expected sterility levels of insects irradiated with the same dose and handling protocols. Thus, together with previous inconclusive investigations, the question arose whether dose really equals dose in terms of biological response, no matter the rate at which the dose is administered. Interestingly, the dose rate effects studied in human nuclear medicine indicated that dose rate could alter dose–response in mammalian cells. Here, we conducted experiments to better understand the interaction of dose and dose rate to assess the effects in irradiated mosquitoes. Our findings suggest that not only does dose rate alter irradiation-induced effects, but that the interaction is not linear and may change with dose. We speculate that the recombination of reactive oxygen species (ROS) in treatments with moderate to high dose rates might minimize indirect radiation-induced effects in mosquitoes and decrease sterility levels, unless dose along with its direct effects is increased. Together with further studies to identify an optimum match of dose and dose rate, these results could assist in the development of improved methods for the production of high-quality sterile mosquitoes to enhance the efficiency of SIT programs.

## Introduction

For the application of the sterile insect technique SIT^1^, insect species targeted for control such as disease-transmitting mosquitoes are mass-reared in a factory. The harmless mosquito males are then separated from blood-sucking females. Subsequently, the male mosquitoes are irradiated with X or gamma radiation to render them reproductively sterile by causing germ-cell chromosome fragmentation that leads to dominant lethal mutations, resulting in imbalanced gametes, the inhibition of mitosis and the ultimate death of the embryo^2^. These sterile males are then transported and released into the area in which the wild populations of the target vector are to be suppressed or eliminated. This strategy, involving sustained sterile male releases, aims to induce sterility in the target insect population and to reduce its density with each generation without affecting the environment and other non-target organisms^1^. For sterile males to succeed in the field, they need to maintain physical quality despite major stressors such as artificial rearing conditions, handling, and radiation exposure. Particularly challenging is the irradiation process where the goal is reliably to achieve near to total sterility whilst keeping the level of off-target, somatic damage as low as possible. Understanding the mosquito's radiation biology and factors that may alter dose–response is crucial to develop standardized irradiation protocols to produce high-quality sterile males. In particular, an acceptable level of consistency in dose–response data is a prerequisite. In this paper, we assess the long neglected topic of dose rate as a variable in insect irradiation, and its implications in the use of nuclear techniques as a method of insect “birth control”. Improving irradiation techniques will contribute to the control of disease vector populations that are responsible for spreading potentially fatal illnesses such as malaria, dengue fever, yellow fever, Zika, and many other arboviruses^3,4^.

Therefore, the question remains whether dose–response changes with the dose rate at which a target dose is administered? The answer can be different depending on the scientific background of the person asked and depending on the angle from which this topic is observed. An entomologist who uses ionizing radiation to sterilize insects for the SIT is likely to be of the opinion that dose–response remains the same, no matter the dose rate^5^. This would make the total absorbed dose the only factor that matters^6^, which is in line with the ‘one-hit’ ionizing event hypothesis^7^. On the contrary, a radiotherapist in oncology could tell you that the dose does not equal dose in terms of biological effects, and dose rate does have a significant impact on irradiation outcome^8–14^. There are many applications of irradiation, all with different goals and desired outcomes. The big difference in these fields of research is the magnitude of the radiation doses that are being applied. Generally, the more complex an organism, the more radiosensitive it is. A human exposed to doses exceeding 10 Gy (10 Sv) will very likely die^15^. Patients undergoing radiotherapy receive doses fractioned into 1–2 Gy at the target site (e.g., a tumor) per treatment, depending on the tissue treated. Insects, however, are very diverse and can tolerate doses of up to 600 Gy, such as some moth (Lepidoptera) species that not only survive such doses but remain partially fertile^16^.

During the last decades, numerous irradiation studies in mosquitoes including some recent research on mosquito SIT have reported widely divergent dose–response results for the same species^17^. Several factors that affect dose–response in mosquito species currently targeted by the SIT (namely *Anopheles arabiensis, Aedes aegypti,* and *Ae. albopictus*) have been investigated at the Insect Pest Control Laboratory IPCL of the Joint FAO/IAEA Centre of Nuclear Techniques in Food and Agriculture, such as handling methods, life stage, pupal age, strain origin, ambient temperature, and atmosphere during irradiation^18,19^. However, additional factors that affect dose response are suspected and require further scrutiny. Dose rate effects have been investigated only sporadically in historic publications, and the topic has mostly been neglected in area-wide integrated pest management programmes that include an SIT component.

### Importance of dose rate

Some studies are available, investigating dose rate effects on lethality^20–22^, insect quality^5,23–25^, and sterility^21,25–28^ but the results have largely been contradictory.

There are on the one hand a series of studies that showed higher damage with higher dose rates when applying a high total absorbed dose. Gonen and Calderon^28^ found that dose rates of 110 Gy min^−1^ administered to males of the cacao moth *Ephestia cautella* induced greater sterility than 28 Gy min^−1^ with total absorbed doses of 200, 300, and 400 Gy. A similar trend was observed in the sawtoothed grain beetle *Oryzaephilus surinamensis*^21^. A study by Haverty and Ware^23^ indicated that mortality of the pink bollworm *Pectinophora gossypiella* increased as the dose rate increased. Jeffries and Banham^23^ demonstrated that increasing dose rate administered to the sawtoothed grain beetle, the confused flour beetle *Tribolium confusum*, and the wheat weevil *Sitophilus granarius* increased detrimental biological effects. Other dose rate dependent negative effects were shown in all life stages of the bruchid beetle *Callosobruchus maculatus*^29^, in food storage pests^21^ and in the codling moth *Cydia pomonella*^20,30^.

Other studies, however, did not corroborate the above results. Ernawan et al.^25^ assessed a series of dose rates in *Aedes aegypti*, but besides affecting some quality parameters such as longevity and mating competitiveness, there was no difference in induced sterility. La Chance^7^ argued that the induced sterility at a given dose should be the same no matter the dose rate, and Hooper^27,31^ found no relationship between dose rate and induced sterility, nor insect quality in two fruit fly species. Subsequently, Collins et al.^5^, tested a dose of 70–75 Gy at increasing dose rates of 5, 7, 26, 57, and 80 Gy min^−1^ on the sterility in the Queensland fruit fly *Bactrocera tryoni* to settle the question of whether dose rate affects dose–response and found no effects on sterility or insect quality. Since the Collins et al. study, the topic has been placed on the backburner and very little has been reported since.

There is, to date, compelling data for and against the one-hit ionizing event hypothesis from an entomological standpoint. This does not imply that one or the other studies may be flawed. However, the studies may have only uncovered pieces of the whole dose rate/dose–response picture. Other factors may influence the degree of dose rate effects. Although hypotheses surround the mechanisms, there has been no research questioning a possible relationship between dose, dose rate, and their combined effects on dose–response. In this study, an attempt was made to control all or most variable factors, including dose rate and dose using attenuators and multiple irradiators (of the same make and model), to investigate the effects of dose rate and define the relationship, if any, between dose, dose rate and induced sterlity, and to uncover the missing pieces to the question “does dose equal dose”.

## Materials and methods

### Biological material

The *Ae. aegypti* strain originated from field collections in Juazeiro, (Bahia), Brazil and was transferred to the IPCL from the insectary of Biofabrica Moscamed, Juazeiro, Brazil in 2016. The *Aedes* strains have been maintained following the “Guidelines for Routine Colony Maintenance of *Aedes* mosquitoes”^32^.

The Dongola strain of *An. arabiensis*, originating from Dongola, Northern State, Sudan, was donated by the Tropical Medical Research Institute, Khartoum, Sudan in 2010 and maintained at the IPCL following the Anopheline mass rearing guidelines^33^.

### Radiation sources and dosimetry

Three Nordion Gammacell 220 (GC 220) irradiators were used with initial dose rates of 1.1, 4.3, and 84 Gy min^−1^. In addition, the dose rate of the 84 Gy min^−1^ irradiator was attenuated with a set of attenuators to 8.3, 26.3, and 38.3 Gy min^−1^. During the experiments the dose rates of each source decayed and the values were noted in each experiment.

#### Calculation of dose rates and dosimetry

Dose rates for each of the three GC220s were assessed and verified using a Farmer type 0.18 cm^3^ free air ionization chamber (10X6-0.18, RadCal Corporation, Monrovia, CA. USA) in conjunction with a digitizer and electrometer (AccuDose Model 9660A) as a reference dosimetry system to measure the dose rate and accumulated dose at a designated reference position. The ion chamber system was calibrated by the John Perry Laboratory (St George’s University Hospital Trust, London) with traceability to the National Physical Laboratory with a calibration factor of 1.0 and uncertainty of 3.3% (k = 2) in the energy range 40–1250 keV.

The irradiation field in the GC220 irradiation chamber has previously been mapped^34^. The samples in this study were placed in the center of the chamber, and in the center of a petri dish within a 2 cm diameter ring. The variation of dose rate within the sample was less than 1%.

The dosimetry system used to verify the dose received by the batches was based on Gafchromic HD-V2 and MD-V3 film (Ashland Advanced Materials, Bridgewater NJ, USA) following the protocol of IAEA^34^. Three films of either HD film (for doses > 50 Gy) or MD film (for < 50 Gy) were packed in aluminium envelopes to protect them from moisture (Aluminum Laminate Detector Pouch FWT-81, Far West Technologies Inc., Goleta, CA, USA) and placed directly above and below the pupae samples. The temperature near the sample and films was measured before and after radiation exposure. Films were read with an optical density reader (DoseReader4, RadGen Ltd, Budapest, Hungary) after 24 h of development.

### Experiment 1: high dose rate vs. low dose rate (2 species, 2 doses, 2 dose rates)

#### Sample preparation and irradiation

Pupae of *Ae. aegypti* (aged 44–48 h) and *An. arabiensis* (aged 20–24 h) were collected and were batched into 3 repetitions of 30 individuals per species, per treatment. Radiation treatments were performed in normoxia, in Petri dishes surrounded by a 4 mm PMMA tube, in either a high activity Gammacell 220 with a dose rate of 84 Gy min^−1^, or in a low activity Gammacell 220 with a dose rate of 1.005 Gy min^−1^. The pupae were contained within a 2 cm diameter ring in the center of the petri dish. Two doses were selected according to the expected dose needed for intermediate sterility and high sterility (> 98%): for *Ae. aegypti* these were 40 Gy and 110 Gy, and for *An. arabiensis*, 90 Gy and 130 Gy. Control pupae were handled identically but were not irradiated. Two biological repetitions each with 3 technical repetitions were performed.

#### Assessment of induced sterility

Following irradiation, males were placed in 15 × 15 × 15 cm Bugdorm cages (MegaView Science Co. Ltd., Taichung 40762, Taiwan) for emergence. 30 virgin females (of the same age) were added to each cage and were allowed to mate for 2 nights, before being bloodfed with fresh porcine blood (*Aedes*) or defrosted bovine blood (*Anopheles*) on 2 consecutive days and were then allowed to oviposit. Egg papers from *Ae. aegypti* were collected, matured (slow-dried over 4 days) and stored for 10 days before hatching. The *An. arabiensis* eggs were collected and hatched the same day. The total number of eggs, (hatched- and un-hatched eggs) were counted for each treatment group using a stereomicroscope to derive the hatch rate. Non hatched eggs were dissected to ensure fertility status. The residual fertility (RF) was calculated as a percentage of the control fertility of each treatment group (RF = HR_tx_/HR_c_ × 100). Induced sterility (IS) was calculated by subtracting the RF from 100%.

### Experiment 2: dose rate response curve (1 species, 1 dose, 6 dose rates)

#### Sample preparation and irradiation

Pupae aged 44–48 h of *Ae. aegypti* were irradiated as described in the previous section “Experiment 1”. All samples were irradiated simultaneously; shorter exposures were performed in the high dose rate GC220 during the longer ones in the low dose rate GC220s. Two biological repetitions each with 3 technical repetitions were performed. The following dose rates were used to irradiate all samples with 20 Gy: 1.1, 4.3, 8.3 26.3, 38.3, and 84 Gy min^−1^. Induced sterility was assessed as described in the previous section “Experiment 1”.

### Experiment 3: Interaction between dose and dose rate (4 doses × 5 dose rates)

#### Sample preparation and irradiation

Pupae aged 44–48 h of *Ae. aegypti* were irradiated as described in the previous section “Experiment 1”. All samples (each with 30 pupae) were irradiated within a given time span; shorter exposures were performed in the high dose rate GC220 during the longer ones in the two low dose rate GC220 irradiators. Three biological repetitions, each with 3 technical repetitions, were performed. The following doses and dose rates were used for the irradiation of samples: 10, 20, 40, 70 Gy and 0.4, 1, 7.8, 24.5, 79 Gy min^−1^, respectively. These dose rates were used due to the availability of gamma sources, and 50%, 70% and 90% attenuators. Induced sterility was assessed as described in the previous section “Experiment 1”.

### Proof of principle: survey on sterilization doses used in SIT projects against *Aedes* spp

To identify possible reasons for the differences reported in the dose needed to achieve sterility in mosquitoes, a survey was conducted in several mosquito SIT research groups in several countries to see what variable factors may account for these differences. The survey included information regarding the biological factors, handling protocols, irradiator type used, characteristics such as the radiation source, and dose rate.

### Statistics

All analyses were performed in R language version 3.2.1^35^. High vs. low dose rate data: Generalized linear mixed models (*glmer* function in lme4 package) were used with radiation dose rate and dose considered as fixed factors and cage (replicates) as a random factor. The full models were checked for overdispersion (using Bolker’s function) and for normality and homogeneity of variances on the residuals. The models were simplified using the stepwise removal of terms, followed by likelihood ratio tests (LRTs). Term removals that significantly reduced explanatory power (p < 0.05) were retained in the minimal adequate model. The significant interactions were analyzed using the *emmeans* function (in package emmeans).

Dose rate response curve: egg hatch rate data were corrected to induced sterility related to the mean control natural sterility and compared between treatments using analysis of variance (ANOVA) and Tukey’s post hoc tests.

A Generalized linear mixed model fit by maximum likelihood was used to analyze the hatch rate data (response variable), using the log of the dose and the hatch rate as fixed effects, and the repeats and technical replicates as random factors^36,37^. Dose rate was used either as a numeric factor or grouped in clusters of similar effect on the hatch rate to account for the non-linear relationship between hatch rate and dose rate. Two groupings were tested, either [0.4; 1; 7.8 Gy min^−1^ or more] or [0.4–1; 7.8; 24.5 Gy min^−1^ or more]. The best model was selected based on the lowest corrected Akaike information criterion (AICc). The likelihood ratio test was used to ascertain the significance of the fixed effects. The R^2^ (coefficient of determination) between the observed and predicted values was used to describe the proportion of variance explained by the selected model^38^.

Student *t*-test was used to compare lowest and highest dose rate effects (DRE) by dose after fertility data was checked for normality and was log transformed.

## Results and discussion

### Experiment 1: low dose rate achieved greater sterility than high dose rate at high doses

Three self-contained Co^60^ gamma-ray irradiators of the same make (Gammacell 220, Nordion Inc., Canada, hereafter called GC220) were used for this experiment with dose rates of 84.07 Gy min^−1^, 4.02 Gy min^−1^ and 1.03 Gy min^−1^. First, we assessed the effects of high dose rate (84 Gy min^−1^) versus low dose rate (1 Gy min^−1^) at two nominal doses (40 Gy and 110 Gy for the mosquito *Ae. aegypti* and 90 Gy and 130 Gy for the mosquito *An. arabiensis*). Irradiated males were mated to untreated females to measure induced sterility in the eggs laid by these females (see M&M for details).

For both species, the low dose rate yielded higher levels of sterility (Supplementary Table S1). In *Ae. aegypti*, a dose of 40 Gy at the high dose rate induced 88.9% sterility, compared to 93.4% following exposure at the low dose rate (P = 0.02). A dose of 110 Gy induced near complete sterility at both dose rates, with only 1 and 2 larvae hatching in one of the 6 repetitions from low dose rate and high dose rate treatments, respectively, and thus could not be compared in a meaningful manner. In *An. arabiensis*, 90 Gy induced 69.6% sterility at the high dose rate, and 87.6% at the low dose rate (P = 0.002). A dose of 130 Gy at the low dose rate achieved complete sterility, while the same dose at the high dose rate only reached 77.0% of induced sterility (IS) (P < 0.05). The differences in the IS following the two treatments was more pronounced in *An. arabiensis*.

The results of this simple experiment were clear, though contradictory to previous reports^20,21,23,28–30^. The low dose rate had a higher impact on the reproductive biology in both species, with higher sterility levels achieved for both medium and high doses (targeting sterility levels of 75% and near 100%). These results were contrary to what was expected based on previously published reports in the literature (cited above), i.e. dose rate effects in insects were generally found to be enhanced as dose rate increases, or the study of Ernawan et al.^25^, that showed no dose rate effects on the sterility of *Ae. aegypti* with a total absorbed dose of 70 Gy. However, they were in line with the reports on “inverse dose rate effects”, the earliest documented in 1979, in HeLa cells by Mitchell et al.^39^.

### Experiment 2: at a low dose, increasing dose rates increased sterility

**Figure 1:**
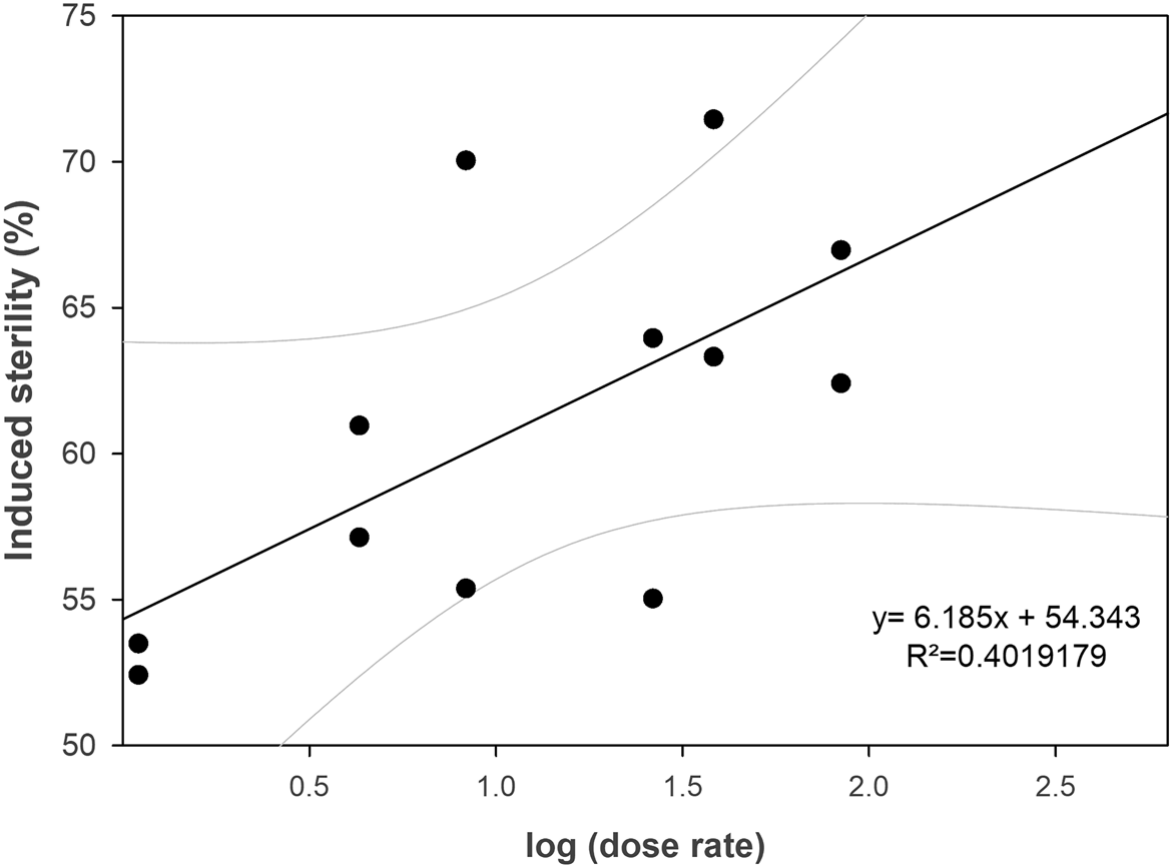
Dose rate response plot showing dose rate effects of *Ae. aegypti* pupae irradiated with 20 Gy on induced sterility in untreated mated females (*P* < 0.02).

As the two doses tested for *Ae. aegypti* induced a very high level of sterility, making it difficult to measure meaningful differences, a lower dose of 20 Gy was selected that was expected to induce around 50% sterility, and tested against a larger range of dose rates. *Ae. aegypti* pupae were used for this experiment and were irradiated in the three available GC220s at dose rates of 1.1 Gy min^−1^, 4.3 Gy min^−1^, and 84 Gy min^−1^ and using 50%, 70%, and 90% dose attenuators (actual attenuation values confirmed as 54.4%, 68.7% and 90.1%), in the high dose rate irradiator, to obtain the following 5 dose rates: 1.1, 4.3, 8.3, 26.3, 38.3, and 84 Gy min^−1^. Results from this assessment showed the opposite of the previous experiment, with irradiation effects on fertility increasing as dose rate increased (Fig. 1), contradicting our previous results, but supporting those of historic reports described above.

The conclusion that radiation effects on the insect cells increase as dose rate increases is consistent with several studies in insects^20–24,28,30,40^. Damage repair mechanisms may be the underlying factor for direct dose rate effect occurrence^41–43^. The increased sterility caused by increasing dose rates could be a side effect of more indirect damage through reactive oxygen species (ROS), and/or due to the higher number of chromosome breaks per exposure, resulting in more broken chromosome ends, which is thought to have an important role in species with holokinetic chromosomes^7^. It is possible to estimate radiation-induced DNA damage using the comet assay, and it has been shown by Shetty et al.^44^ in *Ae. aegypti* that increasing radiation doses result in increased percentage of comet tail DNA. It is questionable whether this assay could pick up small differences in DNA breakage patterns after irradiation at the same dose, but at varying dose rates, but this may be done in future studies.

### Experiment 3: dose rate effects are dose-dependent

Finally, a more in-depth study was carried out to assess potential relationships between dose rate and dose using a series of doses (10, 20, 40, and 70 Gy) administered at a series of dose rates (1.1, 4.3, 8.3 26.3, 38.3, and 84 Gy min^−1^), again using the three GC220s and attenuators.

**Figure 2:**
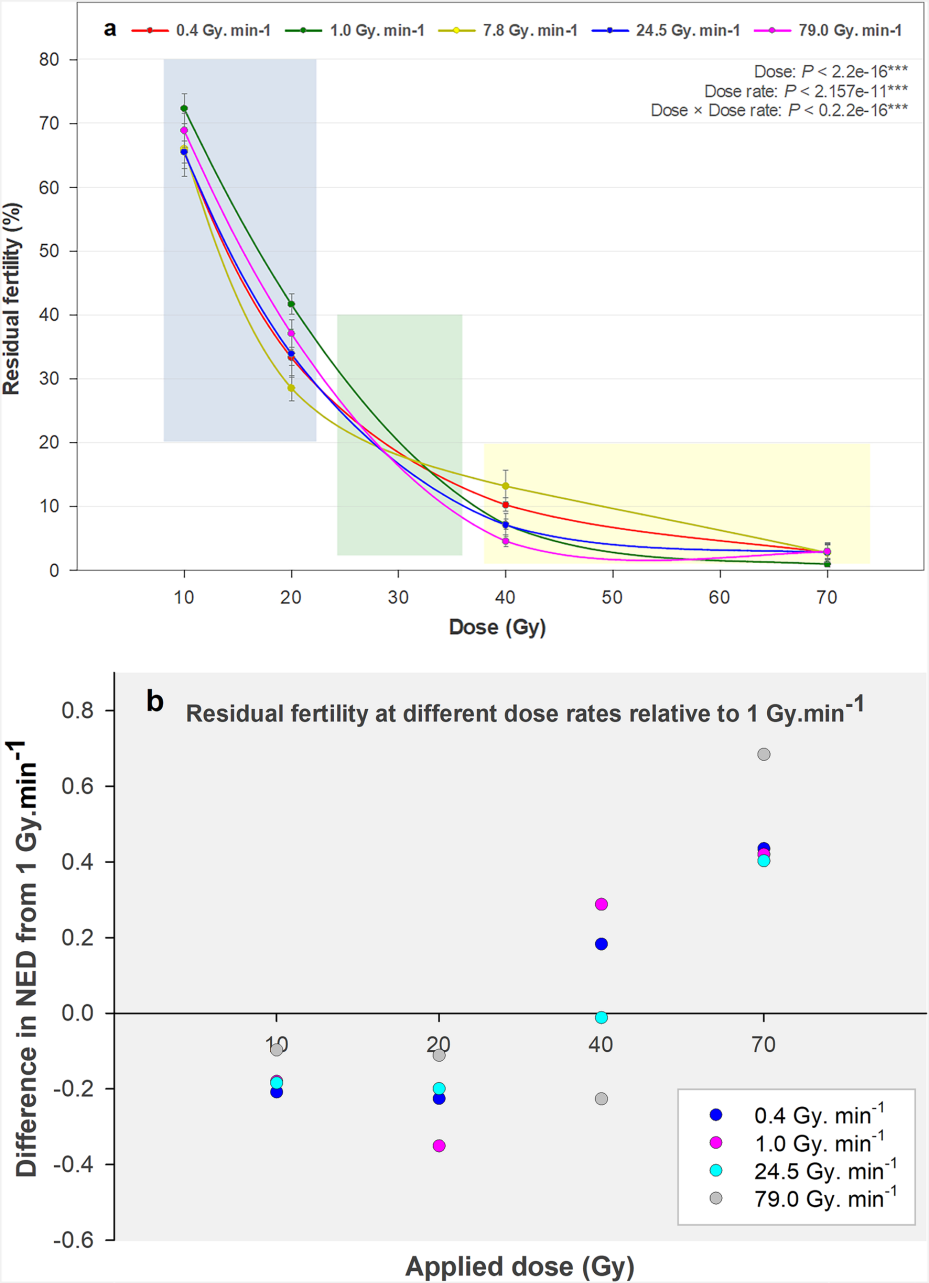
(**a**) Interaction of dose and dose rate: a zone with a positive correlation of the two factors (yellow zone: low doses and increasing dose rates), a negative correlation with inverse dose rate effect (green zone: high doses, with increasing dose rates) and a zone where there are no effects of dose rate (blue zone: mid-range doses). The best model predicting the hatch rate used the log of the dose, the dose rate use as a factor in three groups [0.4; 1; 7.8 Gy min^−1^ or more] and their interaction. It demonstrated that at a low dose, a dose rate of 0.4 Gy min^−1^ or 7.8 or more Gy min^−1^ was more efficient at reducing hatch rate than a dose rate of 1 Gy min^−1^ (p < 10^–3^). The opposite was observed when the dose increased, with a changeover point between 20 and 40 Gy, the dose of 1 Gy min^−1^ becoming the most efficient at inducing sterility (p < 10^–3^). (**b**) Absolute differences in fertility levels (expressed as normalized equivalent deviates, NED) at the various dose rates relative to 1 Gy min^−1^.

The results obtained are summarized in Fig. 2a,b and confirm the results of both preliminary studies. At low doses, radiation effects (observed as a reduction in fertility) increased as dose rate increased. In contrast, at higher doses, these effects (fertility) decreased as dose rates increased, with a turning point where dose rate effects reach a plateau before possibly switching to inverse dose rate effects occurring at doses above 30 Gy (Fig. 2a,b). Absolute differences in fertility levels are also clearly seen when expressed as normalized equivalent deviates, NED) at the various dose rates relative to 1 Gy min^−1^ (Fig. 2b). This result also explains how all historic reports on dose rate effects in insects could be coherent.

As the interaction of dose and dose rate is non-linear, we attempted to depict the relationship by identifying the points where the lowest and highest dose rate effect (DRE) (shown as residual fertility) per dose administered were observed. The results are shown in Supplementary Table S2.

Generally, at lower doses (e.g. 10 and 20 Gy), the DRE increased (sterility increased) as dose rate increased (Fig. 2a). At higher doses (ex. 40 and 70 Gy), DRE decreased (sterility decreased) as dose rate increased. In other words, when identifying which dose rates resulted in the most and least effects on fertility with each dose, these switched when dose increased.

### Proof of principle: survey on sterilization doses used in SIT projects against *Aedes* spp

**Figure 3:**
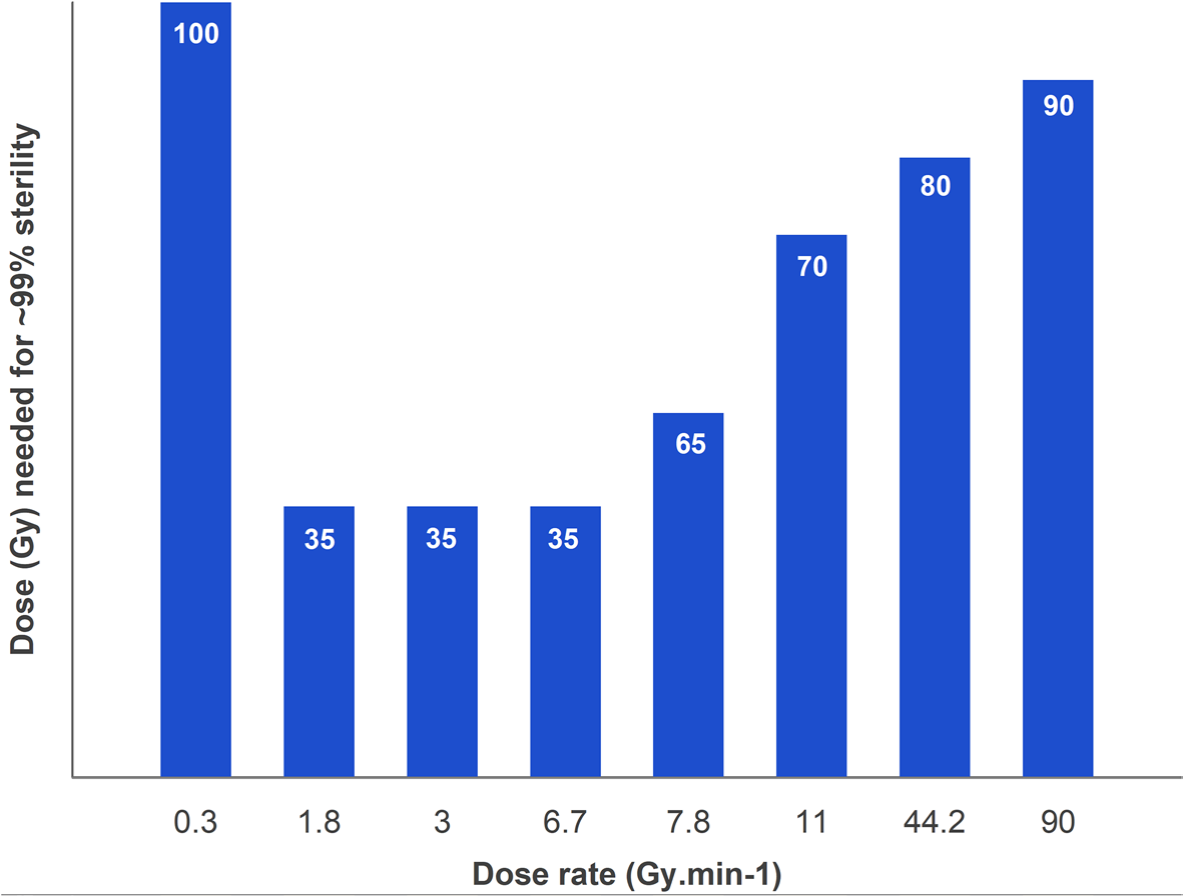
Results from the intercountry survey. Dose required for > 99% induced sterility at different dose rates, regardless of all other parameters and source etc. *Ae aegypti* and *Ae albopictus* data pooled.

A survey was sent to collaborators and colleagues involved in mosquito irradiation and information requested on irradiation materials and methods. The data indicate that the most obvious difference between SIT projects was the irradiation devices used, and their dose rates and energies. Figure 3 summarizes this information and the dose needed to achieve > 99% sterility in *Aedes* mosquito males (regardless of handling methods and other variable factors that may be present). All data were pooled for *Aedes* species (*Ae. aegypti* and *Ae. albopictus* although *Ae. aegypti* is slightly more radioresistant than *Ae. albopictus*), and for source type (Co^60^, Cs^137^, and X-ray) and the doses required for ~ 99% induced sterility were plotted against the dose rate (Fig. 3). Higher doses were required to achieve the same levels of sterility when dose rates increased, except at the very low dose rate of 0.3 Gy min^−1^. Although other external factors such as handling methods, container materials, pupal age etc. may have also contributed to the differences shown, the data provides a conceptual notion that dose rates play a role in mosquito sterilization.

This rough U-shaped graph is a familiar picture and has been observed in some older publications. The viability in the eggs of the rust-red flour beetle (*T. castaneum*) reduced as dose rate increased, but then viability increased again at very high dose rates^40^, giving a V-shaped graph. The authors suggest that the factors that contribute to an increased viability in the embryos at low dose rates (i.e. repair taking place in the cells) are different than those that increase viability at high dose rates (possibly hypoxia induced by high dose rates providing some radioprotection)^40^. Using a hamster model, Vilenchik and Knudson^45^ reported that mutagenic effects in somatic and germ cells are generally reduced as dose rate is reduced, showing a direct dose rate effect, but some cell lines show an inverse dose rate effect at very low dose rates, resulting in a parabolic relationship between dependence of induced mutations and dose rate. In the codling moth (*Cydia pomonella*), dose rate effects were assessed at fixed doses, on larval mortality, pupal mortality, and adult emergence^30^. For all three parameters, the relationship between dose rate and biological effects were not linear, with a positive correlation reaching a limit and then switching to a negative correlation, or vice versa, resulting in U-shaped, or inverted U-shaped graphs. Ernawan et al.^25^ also observed an increase in longevity as well as mating competitiveness as dose rate increased up to a certain threshold, but then these quality parameters decreased again as dose rates were increased further. Although some of these reports insinuated that dose rate had effects partially dependent on dose, the nature of the relationship was not investigated. What is happening in the observed U-effect? We propose that direct and indirect dose effects are affected by dose rate, i.e. inverse dose rate effects.

**Figure 4:**
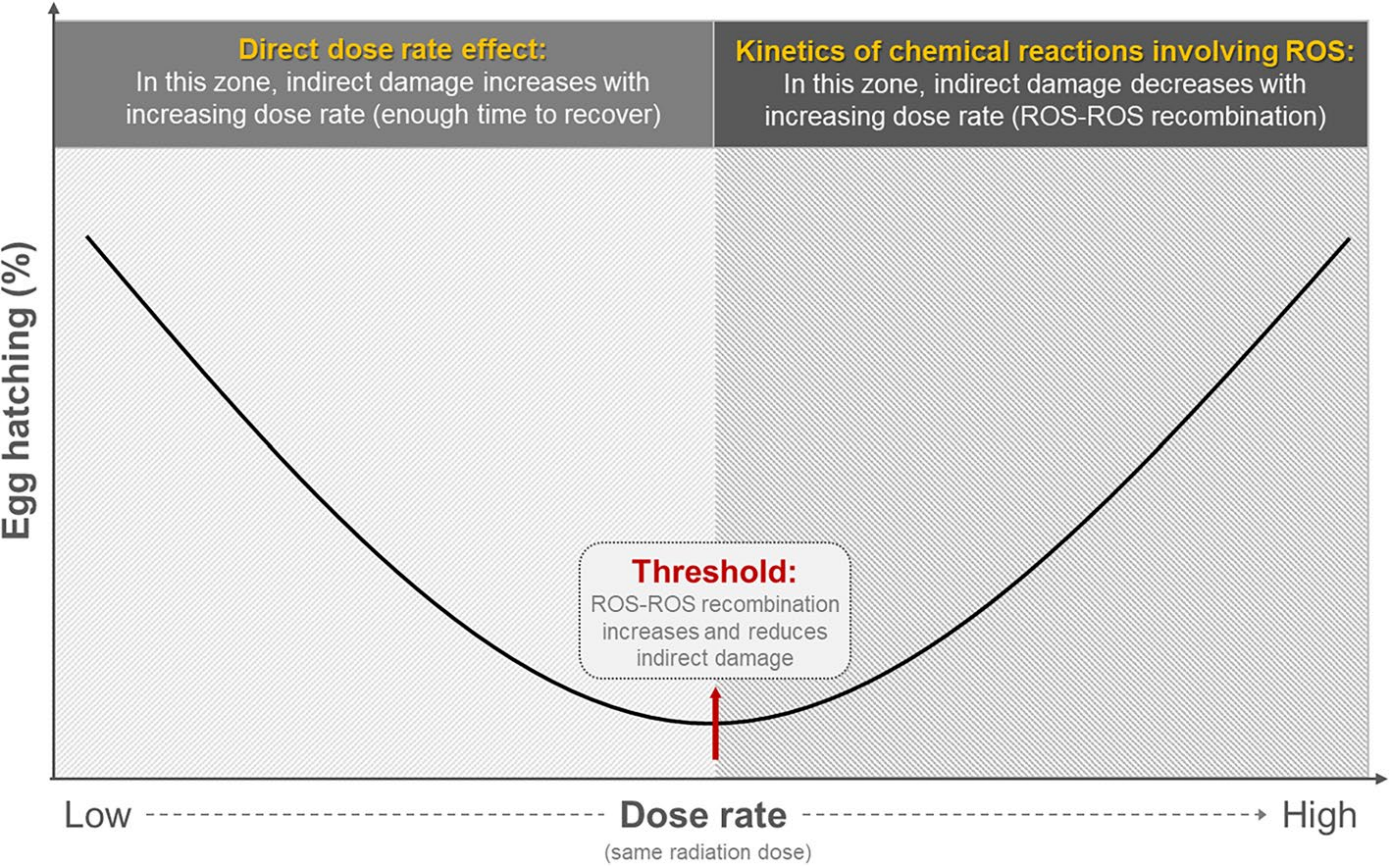
Illustration of the hypothesis for an indirect radiation effects influenced by dose rate, and the differential damage/repair mechanism.

Reactive oxygen species (ROS) can be generated as byproducts of the normal metabolism of oxygen and in response to environmental stress, among other factors. When organisms are exposed to ionizing radiation, ROS can be dramatically generated within cells, primarily through the radiolysis of water, which can cause oxidative stress due to insufficient antioxidative protection, and hence significant damage to DNA and other macromolecules. In our simplistic model (Fig. 4), assuming that radiation dose remains the same, we propose that the higher the dose rate after the threshold, the more ROS–ROS recombination may be observed. The threshold in our current results, in this particular insect was between 20 and 40 Gy, based on Fig. 2a. After this threshold, radiation damage is attenuated by ROS–ROS recombination but can increase if direct damage (by increasing radiation dose) is increased.We base our explanation of the observed inverse dose rate effects on information found in various oncology references whereby we propose that the indirect effect of irradiation is driven by ROS production, which was reduced due to ROS recombination at higher doses and at high dose rates. Another argument, borrowed from the research on FLASH-RT (ultra-high dose rate radiotherapy)^46^, is that irradiation with high dose rates reduces ROS production due to a local transient hypoxia^10^. Under those circumstances (high amounts of ROS being generated in a short time interval with the high dose rate), a high level of sterility could only be achieved by direct effects of irradiation. The increase in sterility could only be achieved by increasing the dose of radiation, which led to more double-stranded breaks through direct effects of radiation^10^.

Additionally, the differential dose response could be due to primary and secondary dose rate effects. Studies from before 1958 on dose rate effects (mainly with *Drosophila* sperm) showed that the accumulation of mutations caused by ionizing radiation was independent of dose rate. This general view was altered post 1958 when Russel^9^, and later Phillips^47^, found fewer mutations in mouse spermatogonia when radiation doses were given at low dose rates, as compared to high dose rates^43^. At this stage, the assumption was that either there was a primary effect of dose rate on the mutation process or secondary effects such as differential killing of cells depending on their intrinsic sensitivity to mutation induction^43^. The theory of a secondary effect was then countered by the investigations by Russel in mice^9^, however the possibilities are so numerous that secondary dose rate effects, although unlikely, cannot be completely dismissed^43^.

The two main theories to explain a primary dose rate effect on the mutation process itself are that (1) mutations are a multi-hit phenomena (likely two-break chromosomal aberrations) and, therefore, need multiple radiation events within a limited time, and (2) that mutations are a one-hit event, but the dose rate dictates the probability of repair of permutational damage within the cells^43^. Most reviews are in favor of the second of the two theories.

The two main mechanisms by which dose rate could affect the repair mechanism are that either there is greater damage to the repair mechanisms when exposed at high dose rates rather than low dose rates, or there is a saturation of the repair mechanism capacity, and there is limited time for it to act when the dose rate is high^43^.

In mice spermatogonia, it was found that the induced mutation rate approaches a lower limit (ca 1/3 of the maximum value) as the dose rate is reduced from 90 to 0.8 rad min^−1^, before it reaches a plateau and stays constant as the dose rate is lowered further to 0.001 rad min^−1^^43,48,49^. In our study, we may also see such a plateau, in which no dose rate effect is seen before the effects are inversed as the dose rate increases or decreases.

In insects, dose rate effects have been investigated in the silkworm^50,51^ where two differing effects were described: in early larval stages, high dose rates produced more mutations than low dose rates (type 1 dose rate effect), whereas in later larval stages, the opposite was observed (type 2 dose rate effect). It was suggested that the type 1 effect can be explained by some influence on the repair mechanism (as suggested for the mouse), and type 2 effects result from selective killing^50^ or from a more persistent inhibition of repair by lower dose rates^51^.

An inverse dose rate effect of ionizing radiation has been reported in human cell response studies^52,53^ and it has been observed in some instances (at higher doses) but not in others (i.e. no dose rate effects in lower doses) within the same study with heterogeneous cell populations^54^. One hypothesis explains this effect on the basis of a “window of sensitivity” in the cell cycle^54,55^, in which the effects of irradiation is enhanced as the exposure time is increased (hence the dose rate is decreased), whereby an enhancement requires “some kind of saturation” where extra hits to a cell result in less than a proportionate increase in the probability of a damage end point^55^. This type of saturation may result in an inverse dose rate effect via a higher incidence of wasted hits during high dose rate exposures, compared to a lower dose rate^55^.

Although it is not fully clear what mechanisms drive the dose rate effects in mosquito dose responses in terms of inducing sterility, there are clues that suggest the existence of a dose rate “region of minimal mutability”^45^ also in mosquito irradiation, suggesting that there may be a possibility to pair dose rate and dose, to optimize the response in a way that high levels of sterility and minimal off-target damage can be achieved, producing a better quality sterile male.

## Conclusions

Our findings may now go some way to explain why some SIT researchers require much higher doses than others to achieve full sterility in the same insect species. Whereas the observed U-shape when pairing dose and dose rate for radiation sterilization has to be accepted as a reality, the mechanisms behind the dose rate dependent effects in inducing sterility in insects remain speculative but very intriguing. The observed take-home message, i.e. there is undoubtedly a dose rate effect when sterilizing mosquitoes and that this effect is dependent on dose, has also been noted with other insects, such as Lepidoptera. As recently observed in the false codling moth *Thaumatotibia leucotreta* programme in South Africa, declining dose rates of a gamma irradiator resulted in lower sterility levels for a total absorbed dose of 150 Gy (Nevil Boersma, personal communication). This epitomizes the absolute need in operational SIT programmes to implement routine and periodic quality control in terms of biological dosimetry when the source of the irradiators in SIT programmes decays over time, or irradiators are reloaded, the configuration of the irradiation canister is changed in any way that would alter the dose rate, or the irradiator itself is exchanged, as the target induced sterility may not be achieved as expected. Failing to implement these quality control measures might result in the release of male insects that are only sub-sterile, which might prolong the programme unnecessarily or in the worst case, even result in programme failure. However, further research is necessary to better understand the dose dependent dose rate effects in insects.

## Supplementary Information

Supplementary Tables.
