# Supplementary material for: Radiation dose-rate is a neglected critical parameter in dose–response of insects
Source: Sci Rep. 2022 Apr 14;12:6242. doi: 10.1038/s41598-022-10027-z (PMC9010456; doi:10.1038/s41598-022-10027-z)
Supplement: Supplementary file 1 — Supplementary Tables. [file 41598_2022_10027_MOESM1_ESM.docx]

## Supplementary file

## Supplementary table 1. Dose response of *Aedes aegypti* and *Anopheles arabiensis* following irradiation with high and low dose-rates

| Species | Dose (Gy) | Dose rate (Gy. min**^-1^)** | IS | SE | Species | Dose (Gy) | Dose rate (Gy. min**^-1^)** | IS | SE |
| --- | --- | --- | --- | --- | --- | --- | --- | --- | --- |
| *Ae. aegypti* | 40 | 84 | 88.94^a^ | 1.767 | *An. arabiensis* | 90 | 84 | 69.62^a^ | 0.026 |
|  |  | 1 | 93.39^b^ | 1.028 |  |  | 1 | 87.57^b^ | 0.031 |
|  | 110 | 84 | 99.86^c^ | 0.138 |  | 130 | 84 | 76.97^c^ | 0.064 |
|  |  | 1 | 99.92^c^ | 0.080 |  |  | 1 | 100.00^d^ | 0.000 |

Dose response measured as induced sterility (IS) with standard error (SE) for *Ae. aegypti* and *An. arabiensis* at mid- and high doses, exposed with high *vs.* low dose rates. Values followed by different letters are significantly different (P < 0.05).

**Supplementary table 2.** The interaction between dose rate and dose seen in residual fertility data:

**
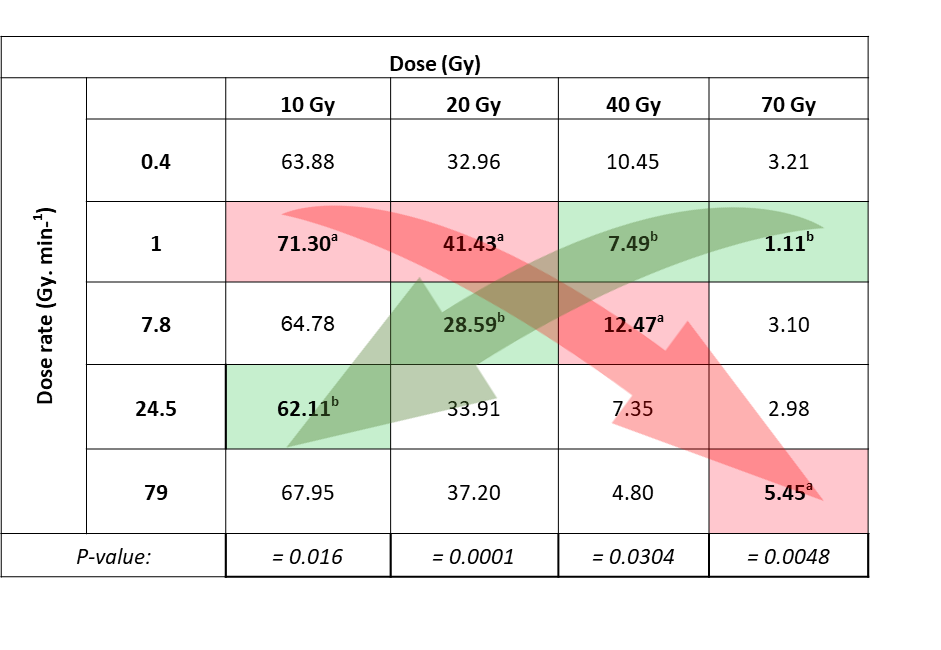
**

Residual fertility values in percent (%). Red represent the lowest dose rate effect (DRE) i.e. (lowest sterility achieved) at a given dose, and green shows the highest DRE (highest sterility achieved) for a given dose. P-values given at the base of each column result from the comparison (t-test) of the low and high DRE values highlighted.
